# Blunt Trauma Abdominal and Pelvic Computed Tomography Has Low Yield for Injuries in More Than One Anatomic Region

**DOI:** 10.5811/westjem.2018.6.37646

**Published:** 2018-07-26

**Authors:** Robert M. Rodriguez, Noah Hawthorne, Shelby P. Murphy, Marcus Theus, David Haase, Chika Chuku, Jason Wen

**Affiliations:** University of California, San Francisco, Department of Emergency Medicine, San Francisco, California

## Abstract

**Introduction:**

Most trauma centers order abdominal and pelvic computed tomography (CT) as an automatically paired CT for adult blunt trauma evaluation. However, excessive CT utilization adds risks of excessive exposure to ionizing radiation, the need to work up incidental findings (leading to unnecessary and invasive tests), and greater costs. Examining a cohort of adult blunt trauma patients that received paired abdominal and pelvic (A/P) CT, we sought to determine the diagnostic yield of clinically significant injuries (CSI) in the following: 1) the abdomen alone; 2) the pelvis alone; 3) the lumbosacral spine alone; and 4) more than one of these anatomic regions concomitantly.

**Methods:**

In this retrospective study, we reviewed the imaging and hospital course of a consecutive sample of blunt trauma activation patients older than 14 years of age who received paired A/P CT during their blunt trauma assessments at an urban Level I trauma center from April through October 2014. Categorization of CSI was determined according to an a priori, expert panel-derived classification scheme.

**Results:**

The median age of the 689 patients who had A/P CT was 48 years old; 68.1% were male; 64.0% were admitted, and hospital mortality was 3.6%. CSI yields were as follows: abdomen 2.2% (95% confidence interval [CI] [1.3–3.6%]); pelvis 2.9% (95% CI [1.9–4.4%]); lumbosacral spine 0.6% (95% CI [0.2–1.5%]); both abdomen and pelvis 0.3% (95% CI [0.1–1.1%]); both the abdomen and lumbosacral spine 0.6% (0.2–1.5%); both the pelvis and lumbosacral spine 0.1% (0.0–0.8%); all three regions – abdomen, pelvis and lumbosacral spine – 0.1% (0.0–0.8%).

**Conclusion:**

Automatic pairing of A/P CT has very low diagnostic yield for CSI in both the abdomen and pelvis. These data suggest a role for selective CT imaging protocols that image these regions individually instead of automatically as a pair.

## INTRODUCTION

With many susceptible organs that are difficult to evaluate by physical exam, the abdomen is often considered the “black box” anatomic region in trauma.^1^ Because of the diagnostic limitations of focused assessment with sonography for trauma (FAST) exam, a computed tomography (CT) is very commonly used to evaluate the abdomen (and pelvis) for injury.^1^ Although they are anatomically distinct, the abdomen and pelvis are traditionally imaged altogether as a single unit in blunt trauma (ordered as abdominal/pelvis [A/P] CT), with lumbar and sacral spine CT included as part of the abdominal and pelvis regions, respectively. Furthermore, A/P CT is often included as part of head-to-pelvis CT (pan-scan) protocol.^2^,^3^

The greater availability of high-speed CT has fueled a dramatic increase in its utilization in acute trauma patient evaluation.^4^,^5^ This rise in use without a concomitant increased prevalence of injury may lead to low CT yields, which in some trauma scenarios approach zero.^6^ Indiscriminant CT use for multiple regions without clear indications for each region can result in harms from over-imaging including costs, unnecessary radiation exposure, and the need to work up incidental findings.^7^–^12^ Beyond the extra costs, a primary concern is the delivery of excess ionizing radiation to radiosensitive tissues, particularly the pelvic organs.^7^–^9^ According to the work of Smith-Bindman et al.,^7^ for every 470 20-year-old women undergoing routine A/P CT with contrast, one woman is predicted to develop a cancer from radiation exposure. Additionally, the need to work up incidental findings, which are very common with A/P CT, may provoke a cascade of excessive testing, including biopsies.^9^–^12^

Nevertheless, the risks and expense of reflexively paired A/P CT may still produce a net benefit if the diagnostic yield in multiple regions is sufficiently high. In this study, we investigated whether the current practice of paired A/P CT in adult blunt trauma evaluation was justified from this standpoint of diagnostic yields. Specifically, we sought to determine the following: 1) the diagnostic yields of A/P CT for clinically significant injury (CSI) in three anatomic regions: the abdomen, the pelvis and the lumbosacral spine; 2) the rates of injury concomitantly in more than one of these three regions; and 3) whether injury seen in one region increases the likelihood of injury in the other regions. We hypothesized that the yield of CSIs distributed in multiple anatomic regions would be very low (< 2%).

## METHODS

### Study Design

In this study, we analyzed data and abstracted charts from the database of our prior study that assessed the yields of CSI with head-to-pelvis CT in blunt trauma evaluation. The study site was an urban Level I trauma center that sees approximately 72,000 patients and 3,800 adult trauma victims per year. The Committee on Human Research approved this study.

### Inclusion and Exclusion Criteria

In the parent study, three abstractors used standard, systematic chart abstraction techniques with frequent audits and checks on inter-abstractor reliability to review the charts of all blunt trauma activation patients older than 14 years of age who received CT imaging during their blunt trauma assessment at this trauma center from April 1, 2014, to October 31, 2014.^13^–^14^ Discordant or ambiguous data were reviewed by the principal investigator. For this analysis, we examined only the 689 charts and data of patients who received A/P CT scans.

**Population Health Research Capsule**

What do we already know about this issue?

Even though they are anatomically distinct, clinicians routinely pair the ordering of abdomen and pelvis computed tomography (CT) in adult blunt trauma patient evaluation.

What was the research question?

What is the diagnostic yield for detecting clinically significant injury (CSI) in both the abdomen and the pelvis in paired abdomen/pelvis CT?

What was the major finding of the study?

The diagnostic yield for CSI in both the abdomen and pelvis is very low. If injury is seen in one region, then there is a higher likelihood of finding injury in the other region.

How does this improve population health?

Our findings suggest a need for more selective, higher-yield CT, which may decrease costs and radiation exposure.

### Data Collection and Processing

Three abstractors collected pertinent patient data using structured abstraction instruments and managed data using Research Electronic Data Capture (REDCap) hosted by the University of California, San Francisco. We transferred data worksheets to Microsoft Excel (2014) for sorting and analysis.

We noted relevant injuries on CT readings in three anatomic regions: the abdomen, pelvis, and lumbosacral spine. To classify injuries, as we have done in previous studies of this topic,^15^–^17^ we convened a panel of 10 associate professor level (or higher) emergency physicians. Each member of the panel independently reviewed a list of traumatic abdominal, pelvis and lumbosacral spine injuries and classified them as either CSI or not. Injuries were classified as CSI if five or more physicians classified it as such. Generally in this classification scheme, injuries were classified as CSI if they required surgical intervention, an interventional radiological procedure, or if they were associated with a blood transfusion. In terms of blood transfusions, we did not distinguish between the index injury and other injuries that could have led to the transfusion. Because of possible need for extended observation, the expert panel also deemed three or more injuries to signify CSI. In terms of location of injury for organs that extend across the abdomen/pelvis border, injuries were analyzed according to where the primary injury was seen on CT. See Table 1 for this classification.

### Outcomes and Data Analyses

Our primary outcome was the yield of CT for CSIs in each of those regions and in various combinations of those regions. We defined yield as the number of patients with at least one CSI to the region or regions of interest divided by the total number of patients receiving A/P CT (n=689). Our secondary outcome was the yield of CT for *any injury* to the three regions and various combinations of those regions. Yield for this secondary outcome was defined as the percentage of the number of patients with at least one injury, regardless of clinical significance, to the region or regions of interest divided by 689. To determine whether CSI in one region was associated with a greater likelihood of CSI in other regions, we calculated odds ratios (ORs) using an online statistics calculator.^18^

## RESULTS

Of the 2,120 eligible patients who presented as blunt trauma activations and had CT during our study period, 689 had A/P CT during their initial work-up. All of these A/P CT were paired; i.e., no patient received isolated abdominal or isolated pelvis CT. A total of 508 (73.7%) of these A/P CTs were ordered as part of head-to-pelvis CT imaging. The median age of patients receiving paired A/P CT was 48 years old (range 15–102 years old), and 469 (68.1%) were male. Refer to Table 2 for patient characteristics.

We list injuries and their classification in Table 3. In Table 4 and Table 5, we present the distributions and yields of CSI injuries and of any injuries. CSIs were seen in the abdomen in 15 (2.2%, 95% confidence interval [CI] [1.3–3.6%]) patients, in the pelvis in 20 (2.9%, 95% CI [1.9–4.4%]) patients, and in the lumbosacral spine in four (0.6%, 95% CI [0.2–1.5%]) patients. CSIs to both the abdomen and pelvis were seen in two (0.3%, 95% CI [0.1–1.1%]) patients, to the abdomen and lumbosacral spine in four (0.6%, 95% CI [0.2–1.5%]) patients, to the pelvis and lumbosacral spine in one (0.1%, 95% CI [0.0–0.8%]) patient, and to the abdomen, pelvis, and lumbosacral spine in one (0.1%, 95% CI [0.0–0.8%]) patient.

Any injury, both clinically significant and clinically insignificant, was seen in the abdomen in 50 (7.3%, 95% CI [5.6–9.4%]) patients, in the pelvis in 64 (9.3%, 95% CI [7.3–11.7%]) patients, and in the lumbosacral spine in 52 (7.5%, 95% CI [5.8–9.8%]) patients. Any injury was seen in both the abdomen and pelvis in 12 (1.7%, 95% CI [1.0–3.0%]) patients, in the abdomen and lumbosacral spine in four (0.6%, 95% CI [0.2–1.5%]) patients, in the pelvis and lumbosacral spine in 13 (1.9%, 95% CI [1.1–3.2%]) patients, and in the abdomen, pelvis and lumbosacral spine in four (0.6%, 95% CI [0.2–1.5%]) patients.

CSI in one anatomic region was associated with an increased likelihood of finding CSI in another region (OR [5.6], 95% CI [1.2–26.7]). Likewise, any injury in one anatomic region was associated with an increased likelihood of finding any injury in another region (OR [3.6], 95% CI [1.8–7.2]).

## DISCUSSION

In this study, we investigated the yield of paired A/P CTs for detecting injuries in multiple anatomic regions in patients who had received blunt trauma. We found that less than 1% of paired A/P CTs revealed a CSI to both the abdomen and pelvis, to both the abdomen and lumbosacral spine, or to both the pelvis and lumbosacral spine and that less than 2% of these scans revealed any concomitant injury, clinically significant or insignificant, to those regional combinations. These low yields, which indicate approximately 345 CTs to detect CSI and 57 CTs to detect any injury in both the abdomen and pelvis, suggest little diagnostic benefit to reflexively pairing CTs of the abdominal and pelvic regions. We also demonstrated that there was a higher chance of seeing injury to either the abdomen or pelvis if there was an injury detected in the other region, a finding similar to that of other studies in which pelvic fractures were shown to be associated with injury in the abdomen.^19^–^21^

CT imaging is not benign and by automatically pairing pelvic CT to an abdominal CT, patients are receiving increased radiation. A typical CT abdomen/pelvis exposes the patient to 15 millisievert (mSv), as opposed to 10 mSv of a CT abdomen alone.^22^ The abdomen and pelvis, including digestive and reproductive organs, are particularly radiosensitive. Exposure to radiation increases the risk of developing malignancies later in life, especially in younger patients.^7^–^9^, ^22^,^23^

Several authors have reported that liberal head-to-pelvis CT imaging has significant utility in the critically ill, poly-trauma patient, and such pan-scan protocols are increasingly used for the evaluation of all adult blunt trauma patients with a concerning mechanism.^3^,^24^,^25^ However, this approach of reflexive head-to-pelvis CT has generated substantial controversy, as experts weigh the balance between not missing clinically significant injuries and attempts to limit costs and radiation exposure.^2^,^25^,^26^ Considering these risks and costs, both the American College of Surgeons and the American College of Emergency Physicians have included the avoidance of reflexive head-to-pelvis CT as part of their Choosing Wisely campaigns.^27^,^28^

Several investigators have proposed guidelines for selective A/P CT in adult trauma patients. However, because these rules require the use of laboratory tests that take time, such as liver function tests, none of these rules has gained wide acceptance in acute trauma evaluation.^29^–^31^ In fact, the majority of trauma patients are not critically ill with multiple sites of severe trauma. The median injury severity score of the 11,477 patients in the NEXUS Chest CT study conducted at eight Level I trauma centers was five.^17^ It is this less critically ill trauma patient population that may benefit the most from selective CT protocols.

Our prior study demonstrated that head-to-pelvis CTs have a low yield for detecting injuries in multiple anatomic regions in patients after blunt trauma, suggesting more selective use of reflexive head-to-pelvis CT.^14^ We also have previously demonstrated that paired CT of the head and neck is common and is a similarly low-yield practice.^17^ Taken with these prior studies, our current findings suggest the need for more selective imaging in certain populations. While the severely injured, poly-trauma patient may still benefit from liberal head-to-pelvis CT protocols, less injured (low-risk) trauma patients may benefit from selective, clinical decision rule-guided (precision) CT, as has been demonstrated by other investigators.^32^,^33^

Overall, our findings suggest that clinicians should consider the uncoupling of abdominal and pelvis CT in lower-risk trauma patients. Toward more selective imaging, clinicians could choose to forego either the pelvis or abdominal portion of CT, depending on trauma mechanisms, physical exam findings and validated clinical decision rules. If a patient’s mechanism and exam suggest that injury is restricted to the abdomen (and not the pelvis), then the CT could be limited to the abdomen region (and vice versa if injury is only suspected in the pelvis). Under such a protocol, our finding that injury found on CT in one region indicates higher likelihood of injury in the other region would suggest that, in those few cases where injury is seen on CT (< 3% for CSI and < 10% for any injury), CT of the other non-imaged region should be enacted. Real-time readings of CT (while patient remains on the CT table) may help prevent back-and-forth trips to the scanner under this strategy. Implementation of such selective CT protocols would require demonstrations of safety (and efficacy) in large multi-center trials.

## LIMITATIONS

The primary limitation of this study is that it was conducted at a single site. Our results may not generalize to other institutions with different patient populations and different trauma CT-ordering practice. We only examined patients over 14 years of age; therefore, our results are not applicable to pediatric populations.

Our retrospective method prevented us from determining the reasons for CT; clinicians may have had strong clinical indicators to order both abdomen and pelvis CT concomitantly. Nevertheless, all CTs were ordered as paired, and it is unlikely that all of these patients had signs of dual abdomen and pelvis trauma.

Regarding the analysis of CT findings, some may question our anatomical location of injuries that may cross from the abdomen into the pelvis (i.e., injuries to the great vessels or sigmoid colon). There is also potential to miss extended injuries to parts of an organ if one were to perform isolated abdominal or pelvic CT.

Finally, clinicians may not agree with our classification of clinical significance and may believe that it is important to detect all (or nearly all) injuries, irrespective of whether these injuries change patient management. Even when considering all injuries, however, the rates of concomitant injury in both the abdomen and pelvis remained very low.

## CONCLUSION

The yield of the current practice of automatically paired A/P CT is low for CSI in more than one anatomic region. When injury is seen in one anatomic region, there is a higher likelihood of having injury in one of the other regions. These data suggest a role for selective imaging protocols instead of the automatic pairing of CT of the abdomen and pelvis.

## Figures and Tables

**Table 1:** Multidisciplinary expert-panel classification of clinical significant injuries.

All abdominal aortic or great vessel injuries
Splenic injury requiring surgical intervention or blood transfusion
Liver injury requiring surgical intervention or blood transfusion
Kidney injury requiring surgical intervention or blood transfusion
Pancreatic injury requiring surgical intervention or blood transfusion
Small or large bowel injury requiring surgical intervention or blood transfusion
Bladder or urethra injury requiring surgical intervention or blood transfusion
Uterine or ovarian injury requiring surgical intervention or blood transfusion
Pelvic bone fracture requiring blood transfusion, stabilization or surgical intervention
Lumbar spine fracture requiring orthotic brace or surgical intervention
Pelvic vessel injury requiring surgical or interventional radiologic procedure or blood transfusion
Three or more injuries in the abdomen or pelvis (chosen as an outcome by the panel’s consensus)

**Table 2:** Patient characteristics (N = 689).

Characteristic	Number (%)
Gender (Male)	469 (68.1%)
Admitted	441 (64.0%)
In-hospital mortality	25 (3.6%)
	Median (Interquartile range)
Age (years)	48 (31,66)
Injury Severity Score	5 (1,14)
Length of hospital stay	4 (2,7)

**Table 3:** Distribution of injuries to abdomen, pelvis, and spine.

Abdominal injuries	Clinically significant	Total
Splenic injury	8	22
Liver injury	4	18
Kidney injury	3	13
Pancreatic injury	0	3
Small bowel injury	1	2
Large bowel/colon injury	0	1
Abdominal aortic injury	3	3
Pelvic		
Bladder/urethra injury	1	4
Uterine injury	0	0
Ovarian injury	0	0
Pelvic bone injury	16	55
Pelvic vessel injury	6	6
Spine		
Lumbar spine injury	4	52

**Table 4:** Yields of abdominal and pelvis computed tomography (N = 689).

Injury detected	Yield for CSI-- # (% [95%CI])	Yield for any injury-- # (% [95% CI])
Injury in abdomen	15 (2.2 [1.3 –3.6])	50 (7.3 [5.6– 9.4])
Injury in pelvis	20 (2.9 [1.9 –4.4])	64 (9.3 [7.3–11.7])
Injury in LS spine	4 (0.6 [0.2 – 1.5])	52 (7.5 [5.8 – 9.8])
Injury in abdomen and pelvis	2 (0.3 [0.1 – 1.1)]	12 (1.7 [1.0 – 3.0])
Injury in abdomen and LS spine	4 (0.6 [0.2 – 1.5])	4 (0.6 [0.2 – 1.5])
Injury in pelvis and LS spine	1 (0.1 [0.0 – 0.8])	13 (1.9 [1.1 – 3.2])
Injury in abdomen, pelvis, and LS spine	1 (0.1 [0.0 – 0.8])	4 (0.6 [0.2 – 1.5])

*LS*, lumbosacral; *CSI,* clinically significant injury; *CI,* confidence interval.

**Table 5:** Frequency of concomitant pelvic and abdominal injury.

Category	CSI frequency (% [95% CI])	Any injury frequency (% [95% CI])
Pelvic injury if has injury to abdomen	2/15 (13.3 [3.7 – 37.9])	12/50 (24.0 [14.3 – 37.4])
Pelvic injury if no injury to abdomen	18/674 (2.7 [1.7 – 4.2])	52/639 (8.1 [6.3 – 10.5])
Abdominal injury if has injury to pelvis	2/20 (10.0 [2.8 – 30.1])	12/64 (18.8 [11.1 – 30.0])
Abdominal injury if no injury to pelvis	13/669 (1.9 [1.1 – 3.3])	38/625 (6.1 [4.5 – 8.2])

*CI,* confidence interval.
